# The Transcriptomic Mechanism of a Novel Autolysis Induced by a Recombinant Antibacterial Peptide from Chicken Expressed in *Pichia pastoris*

**DOI:** 10.3390/molecules27062029

**Published:** 2022-03-21

**Authors:** Dongsheng Wang, Xinjun Yu, Ping Sheng, Guohua Zhang

**Affiliations:** 1 Institute of Biological Resources, Jiangxi Academy of Sciences, Nanchang 330096, China; shengping_1014@163.com (P.S.); zhangguohua2050@163.com (G.Z.); 2 Key Laboratory of Bioorganic Synthesis of Zhejiang Province, College of Biotechnology and Bioengineering, Zhejiang University of Technology, No. 1, Gongda Road, Huzhou 313200, China

**Keywords:** *Pichia pastoris*, RNA-seq, antolysis, mitophagy, MAPK signaling, antibacterial peptide

## Abstract

Autolysis is a common physiological process in eukaryotic cells that is often prevented or applied, especially in yeast expression systems. In this study, an antimicrobial peptide from chicken (AMP) was recombinantly expressed in the *Pichia pastoris* expression system, which induced a series of cellular autolysis phenotypes after methanol treatment, such as the aggregated, lysed, irregular, and enlarged cell morphology, while the cells expressing a recombinant aflatoxin-detoxifizyme (ADTZ) were not autolyzed. A comparative transcriptomic analysis showed that the transcriptomic profiles of cells derived from the autolysis and non-autolysis groups were well discriminated, suggesting that the mechanisms of autolysis were at the transcriptional level. A further differential expression gene (DEG) analysis showed that the DEGs from the two groups were involved mainly in autophagy, the MAPK signaling pathway, transcriptional factors, the central carbon metabolism, anti-stress functions, and so on. In the autolysis group, the cell activity was significantly reduced with the MAPK signaling pathway, the central carbon metabolism was down-regulated, and components of the cytoplasm-to-vacuole targeting (CVT) and mitophagy pathways were up-regulated, suggesting that the autophagy involved in the trafficking of intracellular molecules in the vacuole and mitochondrion contributed to autolysis, which was regulated by transcriptional factors and signal pathways at the transcriptional level. This study provides a theoretical basis for genetic modifications to prevent or utilize cell autolysis in the recombinant protein expression system.

## 1. Introduction

Antimicrobial peptides are a type of small molecular peptides with no toxic side effects (or low toxic side effects) and broad-spectrum antibacterial activity. They are expected to become substitutes for antibiotics and have excellent development and application prospects [1]. Undoubtedly, the recombinant expression of the antimicrobial peptide gene is an efficient method to obtain a large number of antimicrobial peptides [2,3]. At present, in the eukaryotic expression system, the expression of antimicrobial peptides by *Pichia pastoris* is the most common expression host for antimicrobial peptides. Wan et al. successfully expressed active mycelium mycin in *P. pastoris* X33 [2]; Meng et al. successfully expressed a new antimicrobial peptide, PaDef, derived from avocado fruit in *P. pastoris* GS115 [3]. Although there were many studies on the successful expression of antimicrobial peptides by *P. pastoris*, few studies on the large-scale fermentation of antimicrobial peptides by recombinant *P. pastoris* have been reported. One of the reasons for this is that the *P. pastoris* cell that expresses antimicrobial peptides is often unstable after methanol treatments such as autolysis. Thus, cell autolysis is one of the most noteworthy issues to be solved for the recombinant protein expression system [4]. Conversely, the extraction and purification of a recombinant protein may contribute to 80% or more of the total production cost for a gene expression system, and the fact that cells have to be lysed by drastic methods to be recovered can affect the product and the cost [5]. Thus, cell autolysis, after recombinant protein production, is an ideal feature of the expression system for reducing the production cost. A cold-induced promoter driving the endo-beta-1,3-glucanase gene (*eng*) was constructed and transformed into *P. pastoris*, and the transformant underwent autolysis after a cold-shock, which could reduce the protein recovery cost [6]. A NaCl-induced autolysis was performed to isolate and purify glucan from the cell wall of the *P. pastoris* [7]. Although autolysis is an important cellular process which can be prevented or applied in the protein expression system to improve efficiency and reduce cost, the mechanism of this process in *P. pastoris* has not been totally illuminated.

Yeast autolysis has been described as an enzymatic self-degradation of cellular constituents and as the release of different products into the environment. It is proposed to correspond to the dissolution of intracellular organelles that leads to the release of hydrolytic enzymes from the vacuole into the cytoplasm. The mechanism of autolysis in various yeast species, such as baker’s yeast *Saccharomyces cerevisiae* and kefir yeast *Kluyveromyces marxianus*, were illuminated through the transcriptome and proteome, and through biochemical technologies [8,9,10]. Li et al. found that the mitogen-activated protein kinase (MAPK) signaling pathways regulated autolysis through inhibiting the metabolism and disrupting the cell wall in baker’s yeast at the transcriptional level [9]. During the autolysis, in baker’s yeast, the carbohydrate and energy metabolism, amino acid metabolism, and the cell response to various stresses were all repressed by the down-regulation of the corresponding protein at the transcriptional and translational levels [10]. Autophagy is a ubiquitous process in eukaryotic cells that involves the bulk degradation of the cytoplasm and organelles in the vacuole or lysosome. Autophagic transport is performed by autophagosomes, which are double-membrane vesicles containing fractions of the cytoplasm that are targeted to the vacuole. The autophagosome is fused into the vacuolar, and the cytoplasm or organelles are degraded by resident hydrolytic enzymes [11]. More research has focused on the mechanism of autophagy in budding yeast *Saccharomyces cerevisiae*, and the methylotrophic yeasts, *Pichia pastoris* and *Hansenula polymorpha*, leading to the identifications of 32 autophagy-related proteins (ATGs), which functioned at several physiologically continuous steps in autophagy, for example, induction, cargo recognition and packaging, and vesicle formation and breakdown [12]. Autophagy could be either non-selective or selective. Selective autophagy in yeast includes mainly cytoplasm-to-vacuole targeting (CVT), mitophagy, and pexophagy pathways, which degrades the cytoplasm, mitochondria, and peroxisome, respectively [13]. Autophagy genes were regulated at a transcriptional level in response to stress. For example, under starvation conditions, the transcription of the autophagosome marker Atg8/LC3 was rapidly up-regulated in yeast. The expressions of representative ATGs were analyzed, and various new positive and negative regulators of the autophagy pathway were identified [13,14]. Various signal pathways, such as nutrient signaling, MAPK signaling, and target of rapamycin (TOR) signaling, were found to regulate autophagy in yeast [13]. Autophagy may contribute, at least in part, to the outcome of autolysis [6,15]. However, Cebollero et al. discussed the correlation between yeast autophagy and autolysis, and proposed the point in two opposing ways. First, the yeast cells showing increased rates of autophagy would finally lead to accelerated cell death and autolysis. Second, autophagy was essential for survival, and cells defective in autophagy died quickly; thus, it was anticipated that autolysis would be accelerated when such mutant cells were cultured under the carbon-deleted condition [11]. Thus, the relationship between autophagy and autolysis in yeast should be studied further through genetic modification and multi-omics analysis.

*P. pastoris* is one of the most popular eukaryotic expression systems because of its strong promoter of alcohol oxidase I (AOX1), with methanol as the sole carbon resource. However, the cell growth is affected at this time and is closely related to autophagy [16]. When cells encountered hypoxia, a lack of nutrition, organelle damage, abnormal protein accumulation, an invasion of some microorganisms, or chemical treatments, autophagy helped cells survive adversity by degrading intracellular components and maintaining intracellular physiological balance [17]. When *P. pastoris* was cultured on the amino acid-rich methanol medium, autophagy was induced. A transcriptomic analysis showed that the DEGs related to amino acid biosynthesis were up-regulated, and the autophagy induced in *P. pastoris* resulted in a Gcw13-dependent decrease in amino acid uptake, which played a role in the endocytosis of the amino acid permease Gap1 [17]. A methanol-induced high-level production of the Hepatitis B virus surface antigen (HBsAg) was obtained in *P. pastoris*, to which the physiological responses of *P. pastoris* were analyzed through proteome and ultrastructural cell morphology methods. The vacuolar proteases, e.g., APR1, were significantly increased, and the constitutive autophagic processes were observed. Vacuolar proteases were found mainly around peroxisomes, suggesting the induction of the vacuolar degradation pathway. After methanol treatment, the number of cells with spherical vacuoles declined, and cells with irregularly shaped vacuoles increased in number. An electron microscopic analysis revealed the invagination of vacuoles, as is typically observed during peroxisome degradation by microautophagy (micropexophagy) [18]. When the *Aspergillus niger* phytase PhyA was recombinantly expressed in *P. pastoris* under the control of the inducible AOX1 promoter, the MAPK signaling pathway was up-regulated following the methanol induction. Moreover, the improved autophagy pathway after the methanol induction may be related to the pexophagy [19]. However, the induction of autolysis or autophagy by a recombinant antibacterial peptide and its mechanism have never been reported in the *P. pastoris* system. In this study, an antibacterial peptide from chicken was unexpectedly found to induce the autolysis in *P. pastoris*, based on the morphological profile. A systematic transcriptomic technology was applied to illuminate the mechanism of autolysis induced by this antibacterial peptide. This research will deepen our understanding of the relationship between autolysis and autophagy, and provide a theoretical basis for improving the production efficiency and for decreasing the production cost of *P. pastoris* by the genetic engineering of autolysis.

## 2. Materials and Methods

### 2.1. Strain and Culture Conditions

The *Pichia* *pastoris* (*Komagataella phaffii*) X-33 was used to express the antimicrobial peptide (*AMP*) and aflatoxin-detoxifizyme (*ADTZ*) genes. This yeast strain was available from Invitrogen and was maintained in a YPD medium containing 2.0% (*w*/*v*) glucose, 1.0% (*w*/*v*) yeast extract, and 2.0% (*w*/*v*) polypeptone at 30 °C. The *E. coli* strain used in this study was DH5α, kept in our laboratory, and was grown in a Luria broth (LB) medium containing 1.0% (*w*/*v*) tryptone, 0.5% (*w*/*v*) yeast extract, 0.5% (*w*/*v*) NaCl, with a pH of 7.2–7.5, at 37 °C.

### 2.2. Plasmids

The plasmid pPICZα C (Invitrogen, Waltham, MA, USA) was used for the expression of the *AMP* and *ADTZ* genes. The plasmid pUC57-simple for DNA cloning was provided by the Genscript Biotech Corporation (Nanjing, China) Figure S1.

### 2.3. Construction of Plasmids Expressing the AMP and ADTZ Genes

The *AMP* and *ADTZ* genes, derived from the GenBank (accession numbers AY534900.1 and KIJ11450.1), were synthesized and cloned into the plasmid pUC57-simple [20]. The *AMP* gene was then cloned into the pPICZα C to form pPICZα C-*AMP* with the *Xho* I digestion site, AAAAGAGAGGCTGAAGCT sequence coding Kex2 cleavage site, and Ste13 cleavage site at the 5′ end, and the *Xba* I digestion site at the 3′ end. The *ADTZ* gene was cloned into the pPICZα C to form pPICZα C-*ADTZ* with the *Cla* I digestion site at the 5′ end and the *Sac* II digestion site at the 3′ end.

### 2.4. Transformation of P. pastoris

The competent cell preparation was based on Invitrogen’s instructions. Plasmids pPICZα C-*ADTZ* and pPICZα C-*AMP* were linearized by restriction endonuclease *Sac* I, and the linearized DNA products were electrotransformed into the *P. pastoris* competent cell. The transformed cells were plated onto YPD plates with Zeocin and incubated at 30 °C for 3–5 days until colonies appeared. The genomic DNA of randomly selected transformants on the YPD plates was extracted according to the standard protocol (Invitrogen guidelines). Then, the PCR, using 5′ AOX1 and 3′ AOX1 as the primer pair, and using genomic DNA as a template, was performed to validate the positive transformants. The correct transformants were selected for further studies [21].

### 2.5. Induction of the Recombinant AMP and ADTZ in P. pastoris at Bioreactor Scale

The positive transformant was grown on the YPD plate with Zeocin, then transferred into a 500 mL Erlenmeyer flask containing 100 mL YPD liquid medium, and then cultured at 30 °C and 200 rpm overnight as the seed culture. A total of 100 mL of the seed culture was transferred into a BIOTECH-5BG-7000 fermentor (5000 L, Shanghai Baoxing Bio-engineering Equipment, Co., Ltd., Shanghai, China) containing 2000 mL BMGY medium (1.34% *w*/*v* yeast nitrogenous base without amino acids, 1% *w*/*v* yeast extract, 2% *w*/*v* peptone, 1% *v*/*v* glycerin, 10% *v*/*v* 0.1 M potassium phosphate, pH 6.0, 0.00004% *w*/*v* biotin). The fermentation was performed with a temperature of 30 °C, an agitation speed of 200 rpm, and a sterile air or oxygen input volume of 20–40%. After the dissolved oxygen curve increased sharply, which indicated that the glycerol had been exhausted, the methanol was added at an interval of 1.5 h to induce the recombinant AMP and ATDZ productions. During this process, the methanol content in the fermentation broth should not be higher than 1% *v*/*v*.

### 2.6. Sample Collection and Pretreatment

For transcriptomic analysis, the *P. pastoris AMP*-expressing and *ATDZ*-expressing transformants X-33-AMP and X-33-ATDZ, representing the autolysis and the non-autolysis strains, as two groups, were collected at 96 h, respectively. The samples were centrifuged at 5000 rpm and 4 °C for 5 min. The pellets were then washed twice using icy deionized water and preserved at −80 °C for use.

### 2.7. RNA Isolation, Quantification and Qualification

The total RNA of each sample was extracted using TRIzol Reagent (Invitrogen). RNA integrity was assessed using the RNA Nano 6000 Assay Kit of the Bioanalyzer 2100 system (Agilent Technologies, Santa Clara, CA, USA).

### 2.8. Library Preparation for Transcriptome Sequencing

A total amount of 1 μg of RNA per sample was used as the input material for the RNA sample preparations. Briefly, mRNA was purified from total RNA using poly-T oligo-attached magnetic beads. Fragmentation was carried out using divalent cations under elevated temperature in First-Strand Synthesis Reaction Buffer (5X). First-strand cDNA was synthesized using random hexamer primer and M-MuLV Reverse Transcriptase (RNase H). Second-strand cDNA synthesis was subsequently performed using DNA Polymerase I and RNase H. Remaining overhangs were converted into blunt ends via exonuclease/polymerase activities. After the adenylation of the 3′ ends of the DNA fragments, adaptors with hairpin loop structures were ligated to prepare for hybridization. In order to select cDNA fragments of preferentially 370–420 bp in length, the library fragments were purified with the AMPure XP system (Beckman Coulter, Beverly, MA, USA). Then, PCR was performed with Phusion High-Fidelity DNA Polymerase, universal PCR primers, and index (X) primer. At last, PCR products were purified (AMPure XP system) and the library quality was assessed with the Agilent Bioanalyzer 2100 system.

### 2.9. Clustering and Sequencing

The clustering of the index-coded samples was performed on a cBot Cluster Generation System using TruSeq PE Cluster Kit v3-cBot-HS (Illumina, San Dieg, CA, USA) according to the manufacturer’s instructions. After cluster generation, the library preparations were sequenced on an Illumina Novaseq platform, and 150-bp paired-end reads were generated.

### 2.10. RNA-Seq Data Analysis

For quality control, raw data (raw reads) of the FASTQ format were firstly processed through in-house Perl scripts. In this step, clean data (clean reads) were obtained by removing reads containing an adapter, reads containing ploy-N, and low-quality reads from raw data. At the same time, clean data for the Q20, Q30, and GC content were calculated. All the downstream analyses were based on the clean data with high quality.

Reference genome and gene model annotation files were downloaded from the genome website directly. An index of the reference genome was built using Hisat2 v2.0.5, and paired-end clean reads were aligned to the reference genome using Hisat2 v2.0.5. We selected Hisat2 as the mapping tool because Hisat2 can generate a database of splice junctions based on the gene model annotation file and, thus, offer a better mapping result than other non-splice mapping tools.

The mapped reads of each sample were assembled by StringTie (v1.3.3b) in a reference-based approach. StringTie uses a novel network flow algorithm as well as an optional de novo assembly step to assemble and quantitate full-length transcripts representing multiple splice variants for each gene locus.

Feature Counts v1.5.0-p3 was used to count the number of reads mapped to each gene. Then, the FPKM of each gene was calculated based on the length of the gene and the reads count mapped to it. FPKM, or the expected number of fragments per kilobase of the transcript sequence per million base pairs sequenced, considers the effect of sequencing depth and gene length for the reads count at the same time, and is currently the most commonly used method for estimating gene expression levels.

A differential expression analysis of two conditions/groups (two biological replicates per condition) was performed using the DESeq2 R package (1.20.0). DESeq2 provides statistical routines for determining differential expression in digital gene expression data using a model based on the negative binomial distribution. The resulting *p*-values were adjusted using Benjamini and Hochberg’s approach for controlling the false discovery rate. The genes with an adjusted *p*-value < 0.05 found by DESeq2 were designated as differentially expressed genes (DEGs).

Prior to differential gene expression analysis, for each sequenced library, the read counts were adjusted by the edgeR program package through one scaling normalized factor. A differential expression analysis of two conditions was performed using the edgeR R package (3.22.5). The *p*-values were adjusted using the Benjamini–Hochberg method. A corrected *p*-value of 0.05 and an absolute fold change of 2 were set as the thresholds for significantly differential expression. Principal component analysis (PCA), a non-supervised method, was performed to discern the autolysis and non-autolysis groups. Differences between the samples were detected in the PCA score plots, in which each point represented a linear combination of all the DEGs from every sample. The Pearson correlation coefficient was analyzed and visualized by the ggpubr R package.

A gene ontology (GO) enrichment analysis of the DEGs was implemented by the clusterProfiler R package, in which gene length bias was corrected. GO terms with a corrected *p*-value less than 0.05 were considered significantly enriched by DEGs. KEGG is a database resource for understanding the high-level functions and utilities of a biological system, such as a cell, an organism, or an ecosystem, from molecular-level information, especially large-scale molecular datasets generated by genome sequencing and other high-throughput experimental technologies (http://www.genome.jp/kegg/) (accessed on 1 February 2022). We used the clusterProfiler R package to test the statistical enrichment of DEGs in KEGG pathways.

### 2.11. Quantitative Real-Time Reverse Transcription PCR (qRT-PCR)

The expression levels of some DEGs were validated through qRT-PCR. The total RNA was extracted from samples using the methods described in the RNA Isolation section. qRT-PCR was performed with the CFX96 Touch qRT-PCR system (BIORAD, CA, USA). The PCR conditions were as follows: 95 °C for 30 s, 40 cycles at 95 °C for 5 s, and 60 °C for 34 s. The melting curve analyses and agarose gel electrophoresis of the PCR products were conducted to confirm amplification specificity. The relative quantitative method (ΔΔCt) was used to evaluate the relative quantitative variation, and the 18S rRNA gene was used as an internal standard. Every qRT-PCR reaction was performed in triplicate, and the data were normalized using the average for the internal standard.

## 3. Results and Discussion

### 3.1. Effect of the AMP Gene Expression on Cell Morphology

**Figure 1:**
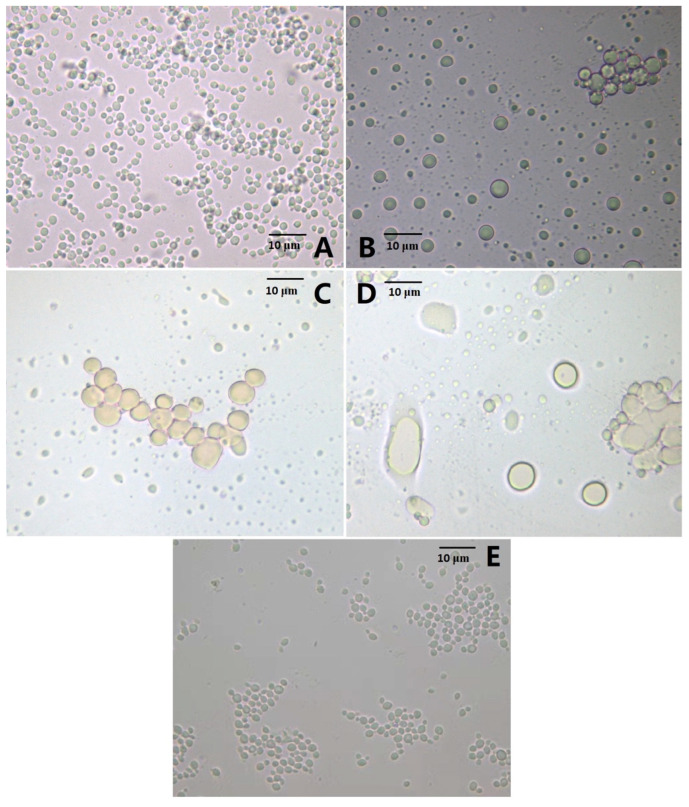
Cell morphology of the *P. pastoris* expressing the antibacterial peptide (AMP). (**A**), the cell before methanol treatment; (**B**), the cell after methanol treatment for 3 h; (**C**), the cell after methanol treatment for 6 h; (**D**), the cell after methanol treatment for 9 h; (**E**), the cell expressing the aflatoxin-detoxifizyme (ADTZ) after methanol treatment for 9 h.

In the previous study, the AMP sequence was identified through bioinformatic analysis based on chicken-expressed sequence tags (ESTs), and the length, Mr, and net charge of the AMP were 148 aa, 16.1 kDa, and +2, respectively. Its high expression levels in different chicken tissues suggested its potency against different pathogens [20]. Thus, the recombinant production of AMP in the *P. pastoris* system is an ideal strategy for industrial purposes. After the transformations of *P. pastoris* X-33 with pPICZα C-AMP and pPICZα C-ADTZ, the transformants *P. pastoris* X-33-AMP and X-33-ADTZ were selected on the zeocin plate. Unexpectedly, after methanol treatment at different times for the recombinant AMP and ADTZ productions, the biomass of X-33-AMP (6.9 g/L) was much lower than those of X-33-ATDZ (58.7 g/L) and X-33 (59.6 g/L). A further microscopic analysis (Figure 1) showed that after the methanol treatment, the number of the X-33-AMP cells was decreased (3 h, Figure 1B), and some cells were aggregated together (6 h, Figure 1C). After the methanol treatment for 9 h, most X-33-AMP cells were lysed, and those that were not broken were irregular and enlarged (Figure 1D). Contrarily, the *P. pastoris* X-33-ATDZ and X-33 cells were not affected noticeably by the methanol treatment (Figure 1E). These results indicated that the recombinant AMP induced by methanol autolyzes the *P. pastoris* X-33-AMP cell, while such autolysis does not occur in the *P. pastoris* X-33-ATDZ and X-33 cells. Antimicrobial peptide could kill human pathogenic bacteria, such as keratinocytes, Staphylococcus aureus, mycobacteria, and E. coli, through inducing cell autophagy [22,23,24]. Meanwhile, various mycotoxin-producing fungi, such as Alternaria, Aspergillus, Penicillium, and Fusarium species were inhibited by the antimicrobial peptide [25]. However, few studies on the autolysis induced by antimicrobial peptides have been reported. Autolysis is a ubiquitous process in eukaryotic cells, in which cells degrade and release intracellular content responding to internal and external stress factors [15]. As is well known, fungal autolysis occurs at the global-cell level and involves various enzymological profiles (proteases, nucleases, and lipid enzymes), signal pathways, and morphological changes [9,26]. The *P. pastoris* morphological changes induced by the AMP in the current study, such as the lesser cell number and the larger cell size, were similar to the previous study. The mechanisms of autolysis in various yeasts, such as Saccharomyces cerevisiae, Kluyveromyces marxianus, and Cryptococcus neoformans, were reported, and the autolysis in yeast was applied in various areas, such as wine making and recombinant protein production [27,28]. *P. pastoris* was used widely as an expression system for recombinant protein production, and the antibacterial peptides derived from various organisms were produced at the scale-up level in this system [29]. A cold-shock-induced prompter of the cctα gene from Saccharomyces cerevisiae was used to drive an endogenous eng gene encoding a putative β-1,3 glucanase in *P. pastoris*, and under cold-shock conditions, the *P. pastoris* cells were autolyzed, releasing the recombinant protein to reduce the extraction and purification cost. However, the systematic mechanism of the autolysis in *P. pastoris* is still lacking. Thus, the transcriptomic profiles of the *P. pastoris* X-33-AMP cells and the *P. pastoris* X-33-ATDZ cells with methanol treatments, representing the autolysis and non-autolysis groups, will be further analyzed and compared to uncover the mechanism of autolysis in *P. pastoris* at the transcriptomic level.

### 3.2. Sequencing and De Novo Assembly of P. pastoris Transcriptome

The total RNAs from the *P. pastoris* X-33-AMP cells and the *P. pastoris* X-33-ATDZ cells, after methanol treatments for 6 h in triplicate, named AP_1, AP_2, and AP_3, and AFX_1, AFX_2, and AFX_3, respectively, were extracted and used to construct RNA-seq libraries. After data cleaning and quality control, the raw reads were processed to a range of approximately 6.30–6.76 G cleaned reads, with the Q20 and Q30 higher than 97% and 92%, respectively (Table S1); thus, the transcriptomic data were qualified for further analysis. A range of 93.81% to 96.3% reads mapped to the reference genome were distributed (Table S2), suggesting that the reference genome selected in this study was satisfactory.

### 3.3. Changes in Transcriptional Profile Induced by the Recombinant AMP in P. pastoris

**Figure 2:**
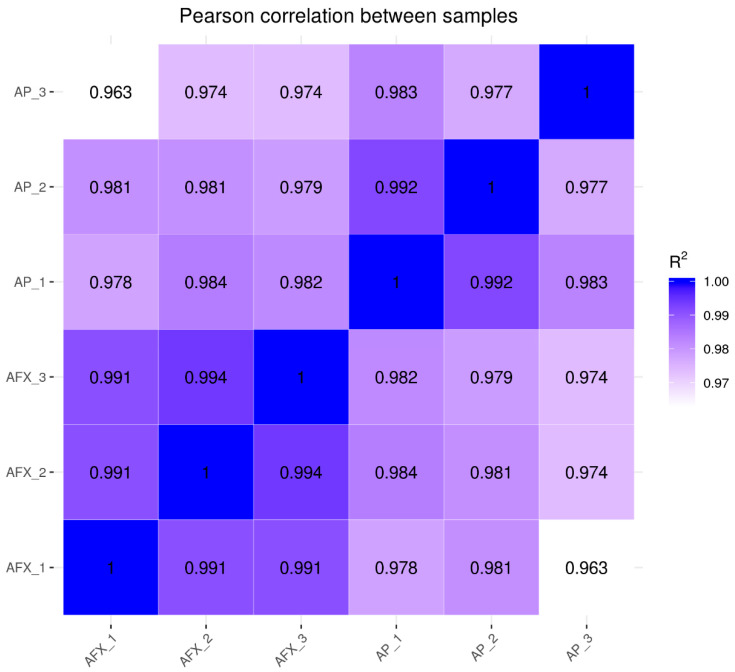
Pearson correlation between the AP and AXP groups. AP, the *P. pastoris* cell expressing the antibacterial peptide (autolysis group)**.** AXF, the *P. pastoris* cell expressing the aflatoxin-detoxifizyme (ADTZ) (non-autolysis group).

**Figure 3:**
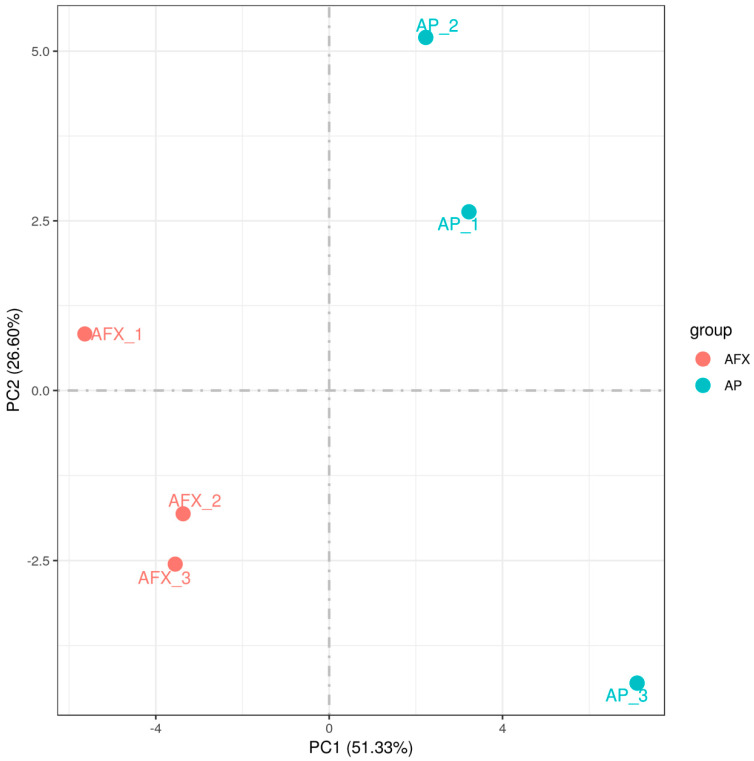
The PCA analysis of the transcriptomic profile derived from the autolysis and non-autolysis groups. AP, the cell expressing the antibacterial peptide (autolysis group); AFX, the cell expressing the aflatoxin-detoxifizyme (ADTZ) (non-autolysis group).

The Pearson correlation coefficients (R^2^) were analyzed to check the sample quality of the biological replicates for each sample. The results showed that the R^2^ of the replicates for each group was higher than 0.977, indicating no significant variation between the biological replicates (Figure 2). Meanwhile, the PCA analysis showed that the transcriptomic profiles between the AP (autolysis) and AFX (non-autolysis) groups varied significantly and were separated clearly at the first component (Figure 3). These results indicated that the mechanism of autolysis induced by recombinant AMP could be explained at the transcriptomic level. Thus, the differential expression genes (DEGs) between the AP and AFX groups were further identified and analyzed to illuminate the mechanism of autolysis in *P. pastoris.*

### 3.4. Overview of the DEGs

**Figure 4:**
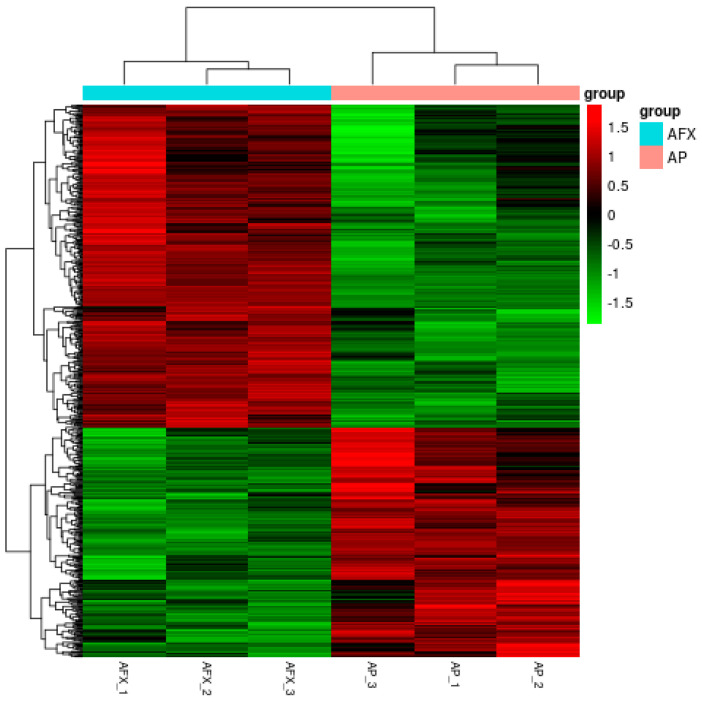
Cluster analysis of the DEGs derived from the AP and AXP groups. AP, the *P. pastoris* cell expressing the antibacterial peptide (autolysis group)**;** AXF, the *P. pastoris* cell expressing the aflatoxin-detoxifizyme (ADTZ) (non-autolysis group); *p*-values < 0.05 were designated as differentially expressed genes (DEGs).

Compared to the AFX group (non-autolysis), there were a total of 617 DEGs in the AP group (autolysis), with 360 down-regulated and 257 up-regulated DEGs. The HCA analysis showed that profiles of the DEGs from the AP (autolysis) and AFX (non-autolysis) groups were discriminated, suggesting that autolysis was involved in the transcriptional levels of these DEGs (Figure 4).

**Figure 5:**
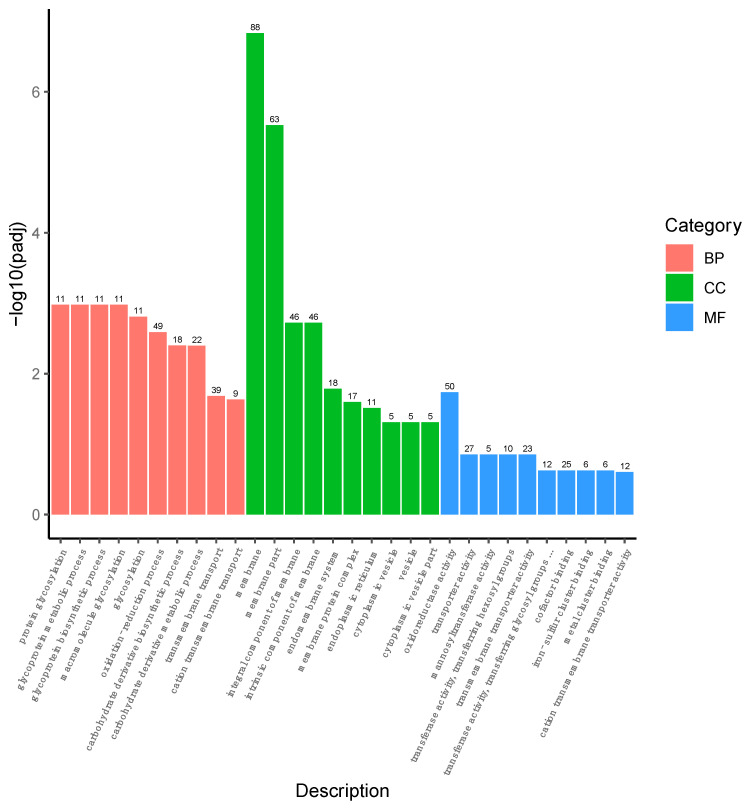
Categorization of GO function for DEGs in *P. pastoris* cells. The ordinate (−log10 (padj)) represents the significance level of GO term enrichment, and the numbers above the graphs indicate the number of genes differentially expressed; *p*-values < 0.05 were designated as differentially expressed genes (DEGs).

A total of 593 DEGs were reannotated into the GO term, and it was found that they were distributed in 30 categories under molecular function (MF), cellular component (CC), and biological process (BP). Among these, 234, 309, and 176 DEGs were distributed in the BP, CC, and MF groups, respectively (Figure 5). In the CC domain, most DEGs were involved in the “membrane”, “membrane part”, “integral component of membrane”, and “intrinsic component of membrane” categories. The highly represented categories in the BP domain were “oxidation–reduction process”, “transmembrane transport”, and “carbohydrate derivative metabolic process”. In the MF domain, “oxidoreductase activity”, “transporter activity”, and “cofactor binding” were the main categories of the DEGs. These results indicated that the DEGs were involved mainly in the membrane, oxidoreductase activity, signal pathway, and molecular interactions.

**Figure 6:**
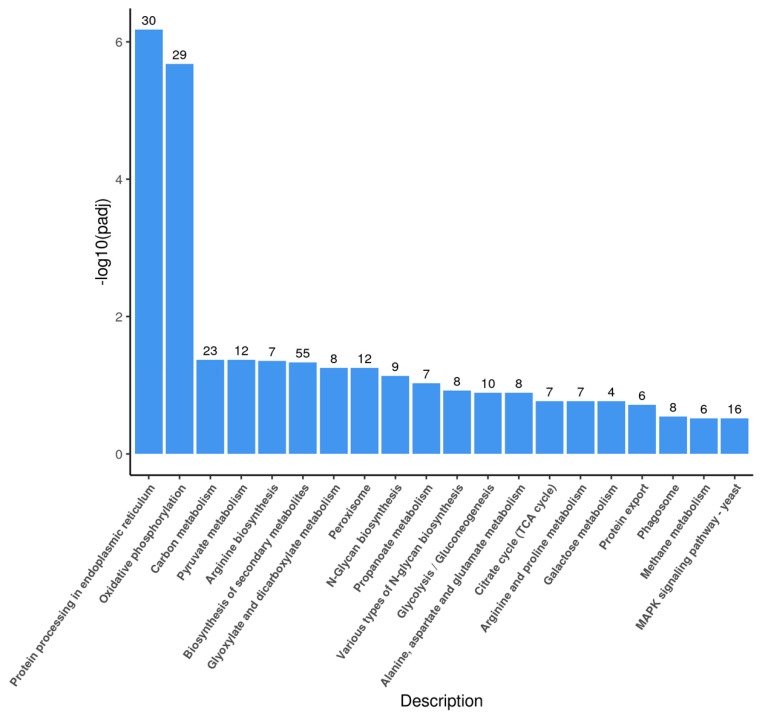
Categorization of Kyoto Encyclopedia of Genes and Genomes (KEGG) function for DEGs in *P. pastoris* cells. The ordinate (−log10 (padj)) represents the significance level of GO term enrichment, and the numbers above the graphs indicate the number of genes differentially expressed; *p*-values < 0.05 were designated as differentially expressed genes (DEGs).

The DEGs were further clustered into the Kyoto Encyclopedia of Genes and Genomes (KEGG) database, and a total of 272 DEGs were assigned to 20 KEGG pathways (Figure 6). The most abundant groups were related to the “biosynthesis of the secondary metabolites” (55/272) category, followed by the “protein processing in endoplasmic reticulum” (30/272), and “oxidative phosphorylation” (29/272) categories.

### 3.5. DEGs Related to the Autolysis in P. pastoris

As shown in Table 1, various DEGs comparing the AFX and AP groups were involved in the MAPK signaling pathway. The expression levels of NAD-dependent dehydratase, mitogen-activated protein kinase, and protein kinase C were decreased by 0.45-, 0.28-, and 0.25-fold in the AP group (the autolysis group), compared to the AFX group (the non-autolysis group). In the plant pathogen fungus *Colletotrichum gloeosporioides*, a mitogen-activated protein kinase (MAPK) CgMk1 could be phosphorated by its upstream components, CgSte50, MAPKKK CgSte11, and MAPKK CgSte7, and the CgMk1-deficient mutant resulted in the loss of the appressorium formation, invasive growth, and pathogenicity, and the cell autolyzed with increased septum formation in the hyphae [30]. In the baker’s yeast *Saccharomyces cerevisiae*, the DEGs under the autolysis condition were significantly enriched in the MAPK signaling pathway, and they were all expressed in a down-regulated form. This study suggested that the MAPK signal pathway plays an important role in the yeast autolysis process, and that autolysis inhibits the activity of yeast cells in energy production and utilization [9]. In the current study, almost all of the DEGs related to the MAPK signal pathway were inhibited during the autolysis of the *P. pastoris*, indicating that the MAPK signal pathway regulated the autolysis process closely, and that the cell activity was inhibited at the global level during autolysis. Meanwhile, the casein kinase gene was induced by 0.23-fold in the AP group, compared to the AFX group, suggesting that this gene was induced by cell autolysis (Table 1). Casein kinase is a ubiquitous and conserved phosphate transferase that is critical for the growth and development of eukaryotic cells. In *Penicillium oxalicun*, Lei et al. found that different casein kinase mutants reduced expressions of transcription factors, delayed autolysis in a carbon starvation medium, and decreased productions of cellulase and amylase, compared with the wild type. These results indicated that casein kinase was an essential component in the signal pathway and played an important role in autolysis and cell degradation at a transcriptional level [31].

The transcription factor is a linkage between the signal pathway and downstream genetic regulation; thus, it is a point among DEGs to analyze. As shown in Table 1, the TBP-associated factor (TFA) and the bZIP transcription factor were identified as two DEGs with a 0.22-fold decrease and 0.27-fold increase in the autolysis group, compared to the non-autolysis group. A mutant with the bZIP-type TF gene deleted in the pathogenic fungus *Metarhizium robertsii* (MBZ1) impaired its growth and comidiogenesis, and autolysis was induced in this mutant [32]. A bZIP-type TF FlbB in *Aspergillus nidulans* fungi was found to regulate autolysis under a high sorbitol or sucrose concentrations, revealing its role in vegetative growth [33]. In the current study, it has been found, for the first time, that two transcription factors regulate the autolysis in *P. pastoris*, which validates that the mechanism of the autolysis induced by the AMP is at the transcriptional level. These TFs may be the genetic targets in further TF engineering for applying autolysis in industrial areas.

A series of autolysis genes, such as the vesicle transport soluble *N*-ethylmaleimide-sensitive factor attachment protein receptors (SNARE) protein, vacuolar proteinase, vacuolar sporting-related protein, clathrin assembly protein, and heat shock protein 70, were identified as the DEGs, and their transcriptions were all up-regulated in the AMP group (autolysis), comparing with the ATDZ group (non-autolysis) (Table 1). The SNARE proteins mediate fusions between vesicles or a vesicle and the target membrane, which results in exocytosis; thus, the SNARE protein plays an important role in the recombinant protein secretion of *P. pastoris* [34]. The SNARE protein also involves the formation of double-membrane-bound organelles, called autophagosomes, in *P. pastoris*, which aids in the degradation of cellular components through fusion with lysosomes [35]. Vacuolar proteinase and vacuolar sporting-related proteins are related to vacuolar fusion, protein sorting into the vacuole, and proteolysis in the vacuole [36]. The results indicated that some cellular macromolecules were transported into the vacuole and degraded in the vacuole during the *P. pastoris* autolysis. The clathrin assembly protein performs critical roles in shaping rounded vesicles in the cytoplasm for intracellular molecule trafficking. The clathrin-coated vesicles (CCV) selectively sort cargo for multiple membrane traffic pathways, and regulate the initiation of autophagy. Thus, the increased expression level of the clathrin assembly protein in the autolysis group may be used to induce the CCV-mediated autolysis in the *P. pastoris* expressing the *AMP* gene. As shown in Table 1, leucine zipper/EF-hand-containing transmembrane protein 1 (LETM1) was down-regulated by 0.28-fold in the AF (autolysis) group, compared to the AFX (non-autolysis) group. The LETM1 protein is a conserved eukaryotic protein with a transmembrane domain, and it is located in the mitochondrial inner membrane. It governs the mitochondrion ion channel and is involved in mitochondrial respiration. Moreover, the remarkable role of the LETM1 in mitochondrion-mediated cell autolysis has been summarized [37]. It is worth mentioning that the down-regulation of LETM1 induced autophagy in colorectal cancer (CRC) cells, and the autophagy inhibitor 3-methyladenine reversed the inhibitory effects of LETM1 silencing on proliferation and stemness. A further analysis indicated that the suppression of LETM1 increased the levels of reactive oxygen species (ROS) and mitochondrial ROS, activated the AMP-activated protein kinase (AMPK)/mammalian target of rapamycin (mTOR) and, thus, initiated autolysis [38]. In our current study, the LETM1 protein was significantly suppressed, and the AMPK pathway was promoted in the AP (autolysis) group, compared to the AXP (non-autolysis) group, indicating that the suppression of LETM1 induced the AMPK pathway and initiated autolysis in *P. pastoris*.

*P. pastoris* uses methanol as the sole carbon resource for growth and recombinant protein production, and methanol is partially oxidized into CO_2_; the rest is converted into glycerone-P, which subsequently enters into central carbon metabolism, energy metabolism, and amino acid biosynthesis [39]. Thus, the central carbon metabolism plays an essential role in the cell autolysis of *P. pastoris*. As shown in Table 1, various DEGs, such as aldehyde dehydrogenase, methanol oxidase, isocitrate lyase, malate synthase, acetyl-CoA synthase, and citrate synthase, were involved in the central carbon metabolism, and the expression levels were significantly decreased in the AF (autolysis) group, compared to the AXF (non-autolysis) group. As a result, the cells from the autolysis group had relatively weak cell viability, and the initiation of autolysis, induced by the recombinant AMP, was improved. Zou et al. found that autophagy was induced in *P. pastoris* when cells were grown in an amino acid-rich methanol medium, and that the autophagy may be due to a GPI-anchored protein (Gcw13)-dependent decrease in amino acid uptake during methanol adaptation [17]. The *P. pastoris* mutant with the two deleted catabolite repressor genes *MIG1* and *MIG2* realized the derepression of the alcohol oxidase (AOX) when grown in glycerol. Most of the autophagy-related genes (ARG) were down-regulated in the Δ*mig1*Δ*mig2* mutant; as a result, that the autophagy was ameliorated [40].

To validate the RNA-seq results, a total of 19 DEGs were selected from the four groups and amplified using the qRT-PCR technology. As shown in Table 1, the expression patterns of the selected DEGs were consistent with those of the transcriptomic profiles, confirming the reliability of the RNA-seq results.

## 4. Conclusions

*P. pastoris* is a popular system for producing recombinant antimicrobial peptides derived from various organisms with great value. However, autolysis is a problem to be solved for improving productivity, or a potential to be applied for reducing production cost. In this study, it was found that an antimicrobial peptides (AMP) recombinantly expressed in *P. pastoris* can induce autolysis after methanol treatment, such as the aggregated, lysed, irregular, and enlarged cell. The transcriptomic profiles between the autolysis and non-autolysis cells were well discriminated, suggesting that the induction of autolysis was at a transcriptional level. The DEGs were involved mainly in autophagy, the MAPK signaling pathway, the transcriptional factor, the carbon metabolism, and anti-stress functions. In the autolysis group, the cell activity was decreased by down-regulating the MAPK signaling pathway and the central carbon metabolism, while the autophagy pathway was improved. The autophagy is involved mainly in the assembly, trafficking, and degradation of intracellular molecules in a vacuole and mitochondrion, and is regulated by signal pathway and transcriptional factors at the transcriptional level. This study provides a theoretical basis for the genetic modification of yeast cell autolysis.

## Figures and Tables

**Table 1:** Variations in the expressions of key genes in the autolysis and non-autolysis *P. pastoris* cells.

Group	Gene ID	Description	FPKM			RT-PCR log2-Fold Change
			AFX	AP	log2-Fold Change	
Autophagy	PAS_chr3_1085	Vesicle transport SNARE protein	1044 ± 190.3	1362 ± 349.3	0.38	0.32
	PAS_chr1-4_0548	Vacuolar proteinase	8502 ± 100.4	10,225 ± 2434.9	0.27	0.34
	PAS_chr2-2_0264	Vacuolar sporting-related protein	690 ± 49.3	844 ± 29.7	0.29	0.22
	PAS_chr4_0998	LETM1 homolog	732 ± 149.2	605 ± 32.5	0.28	0.42
	PAS_chr3_1047	Clathrin assembly protein	348 ± 50.4	449 ± 78.5	0.37	0.45
	PAS_chr3_0230	Heat shock protein 70	105,004 ± 10,357.2	124,125 ± 2985.4	0.2	0.14
MAPK signaling pathway	PAS_chr3_0449	NAD-dependent dehydratase	6376 ± 190.4	4661 ± 684.3	0.45	0.49
	PAS_chr4_0530	Mitogen-activated protein kinase	1990 ± 243.2	1445 ± 684.2	0.28	0.22
	PAS_chr2-1_0124	Protein kinase C	4423 ± 146.3	3721 ± 283.5	0.25	0.22
	PAS_chr2-1_0358	Casein kinase	1440 ± 424.2	1692 ± 168.4	0.23	0.42
Transcriptional factor	PAS_chr2-1_0142	TBP-associated factor	1188 ± 59.3	985 ± 38.5	0.27	0.46
	PAS_chr4_0204	bZIP transcription factor	4913 ± 484.2	5729 ± 258.6	0.22	0.25
Carbon metabolism	PAS_chr4_0470	Aldehyde dehydrogenase	6260 ± 243.1	2527 ± 864.3	1.3	2.52
	PAS_chr4_0152	Methanol oxidase	64,007 ± 734.2	35,007 ± 2698.5	0.83	0.92
	PAS_chr1-4_0338	Isocitrate lyase	11,657 ± 985.2	6844 ± 49.3	0.77	1.03
	PAS_chr4_0191	Malate synthase	64,415 ± 484.2	45,077 ± 5483.2	0.51	1.42
	PAS_chr3_0403	Acetyl-CoA synthase	2023 ± 354.2	1435 ± 235.2	0.5	0.94
	PAS_chr1-1_0475	Citrate synthase	38,285 ± 6323.2	28,902 ± 1984.3	0.41	0.63
Anti-stress	PAS_chr1-1_0433	Mitochondrial thiol peroxidase	4593 ± 354.2	5692 ± 254.6	0.29	0.37

Data are given as means ± standard deviation, *n* = 3; *p*-values < 0.05 were designated as differentially expressed genes (DEGs); AFX, the cell expressing the aflatoxin-detoxifizyme (ADTZ, non-autolysis group); AP, the cell expressing the antimicrobial peptides from chicken (AMP, autolysis group).

## Supplementary Materials

The following supporting information can be downloaded at: https://www.mdpi.com/article/10.3390/molecules27062029/s1, Figure S1: The diagram of the plasmid pPICZα C-AMP; Table S1: Quality control of data after processing; Table S2: The total reads and map percentages of data.

## Sample Availability

Samples of the compounds are not available from the authors.
