# Supplementary material for: The Transcriptomic Mechanism of a Novel Autolysis Induced by a Recombinant Antibacterial Peptide from Chicken Expressed in Pichia pastoris
Source: Molecules. 2022 Mar 21;27(6):2029. doi: 10.3390/molecules27062029 (PMC8955930; doi:10.3390/molecules27062029)
Supplement: Supplementary file 1 [file molecules-27-02029-s001.zip › molecules-1612407-supplementary.pdf]

Table S1 Quality control of data after processing.

sample	raw_reads	clean_reads	clean_bases	Q20	Q30	GC content
AFX_1	42818082	41966864	6.3G	97.75	93.14	44.25
AFX_2	45678016	44611564	6.69G	97.81	93.36	44.2
AFX_3	44832542	43708314	6.56G	97.75	93.13	44.16
AP_1	44408450	43376640	6.51G	97.58	92.81	44.16
AP_2	45861312	45063184	6.76G	97.88	93.46	44.13
AP_3	43510568	42433742	6.37G	97.41	92.39	43.89

Table S2 The total reads and map percentages of data.

sample	total_reads	total_map
AFX_1	41966864	39455808(94.02%)
AFX_2	44611564	41852040(93.81%)
AFX_3	43708314	41072327(93.97%)
AP_1	43376640	41667068(96.06%)
AP_2	45063184	43374096(96.25%)
AP_3	42433742	40861704(96.3%)

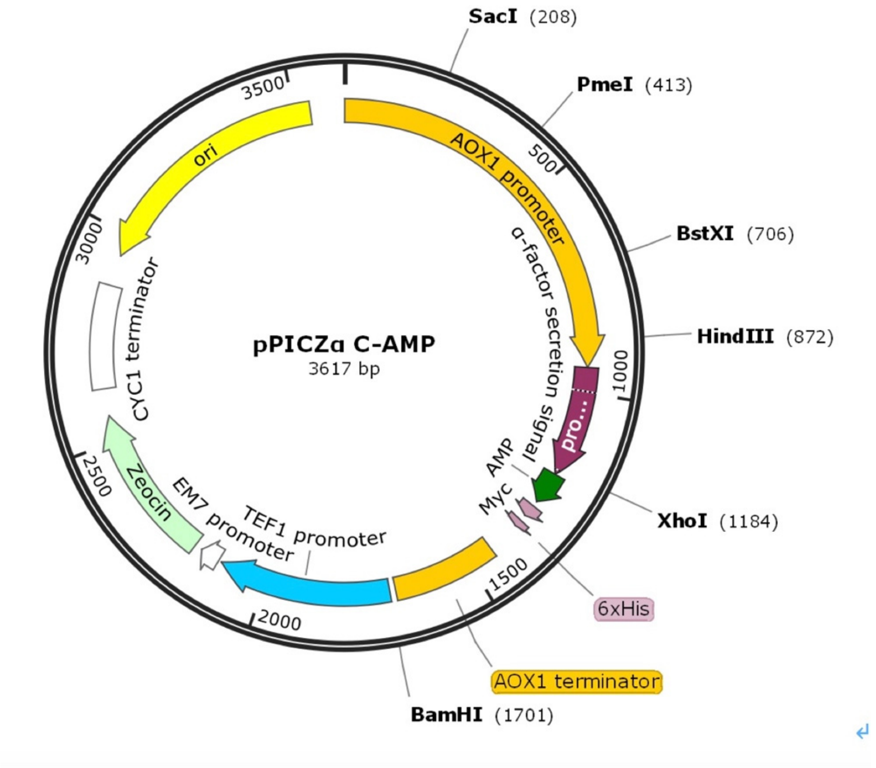

Figure S1 The diagram of the plasmid pPICZα C-AMP.

The codon-optimized AMP gene sequence for *P. pastoris*

ATGCTATCTTGTTGGGTTCTGTTGCTAGCATTGTTAGGCGGAGCCTGTGCCCTGCCTGC
TCCATTGGGTTACTCTCAAGCTTTGGCACAAGCCGTCGATAGTTACAACCAACGACCC
GAGGTTCAAAACGCTTTCAGATTGTTATCCGCTGACCCTGAGCCAGGACCTAATGTGC
AATTGAGTTCTCTGCATAACTTGAACTTTACCATAATGGAGACAAGATGTCAAGCCAGA
TCAGGTGCCCAGTTGGATTCCTGTGAGTTCAAAGAAGATGGATTAGTGAAAGATTGTG
CAGCACCAGTTGTTTTACAAGGTGGTCGTGCTGTCTTAGACGTAACCTGTGTTGATTCA
ATGGCCGACCCTGTTCGTGTTAAACGTGTTTGGCCTTTGGTGATCCGTACCGTTATCGC
TGGTTACAACTTGTACAGAGCTATTAAAAAGAAATGA
